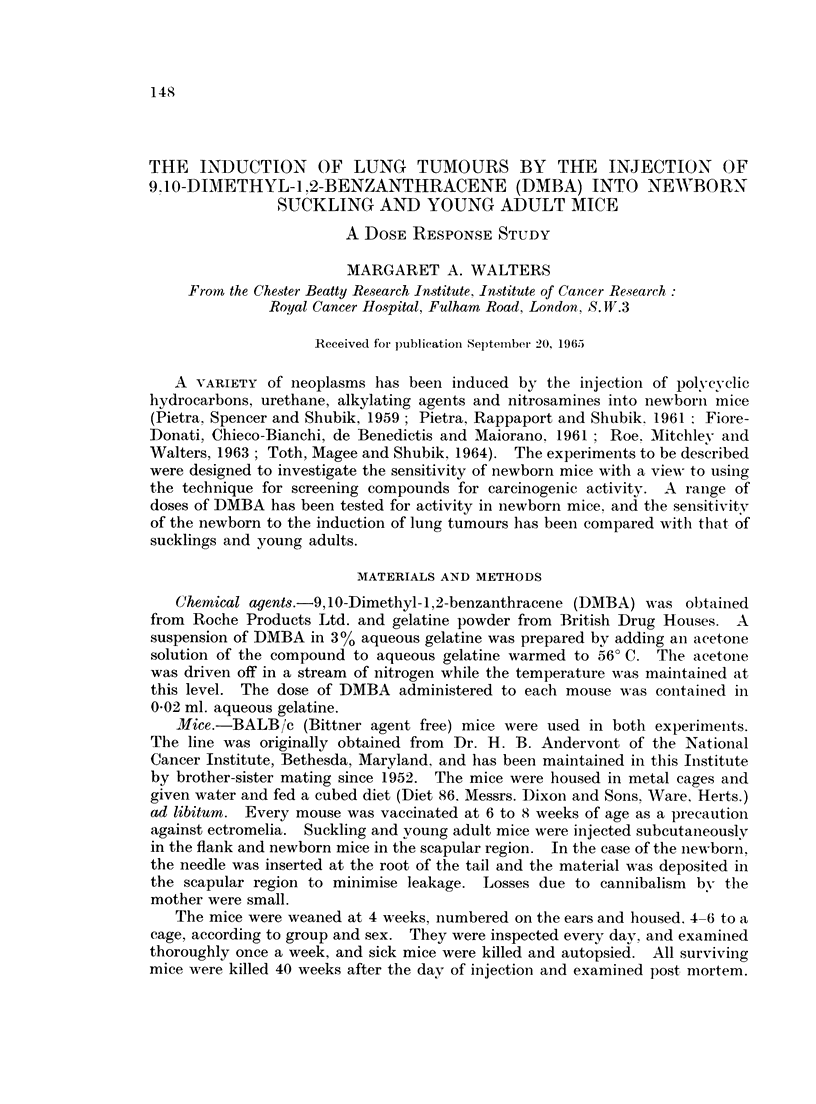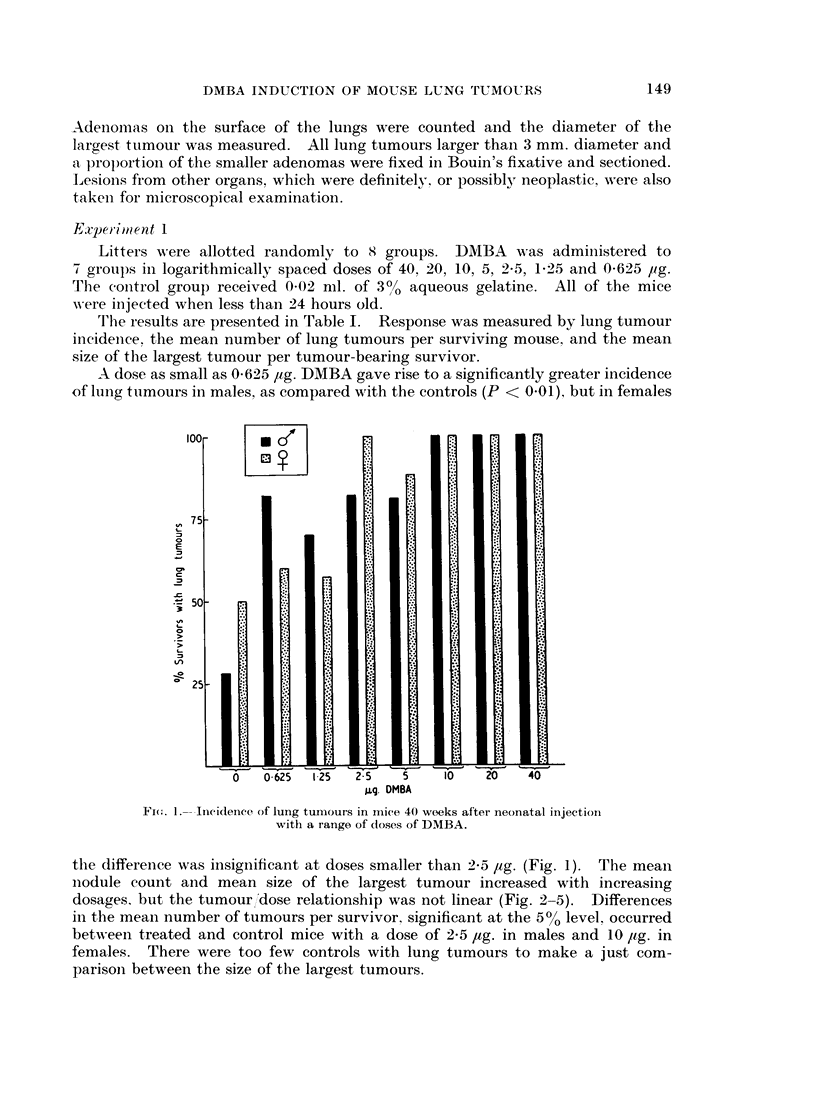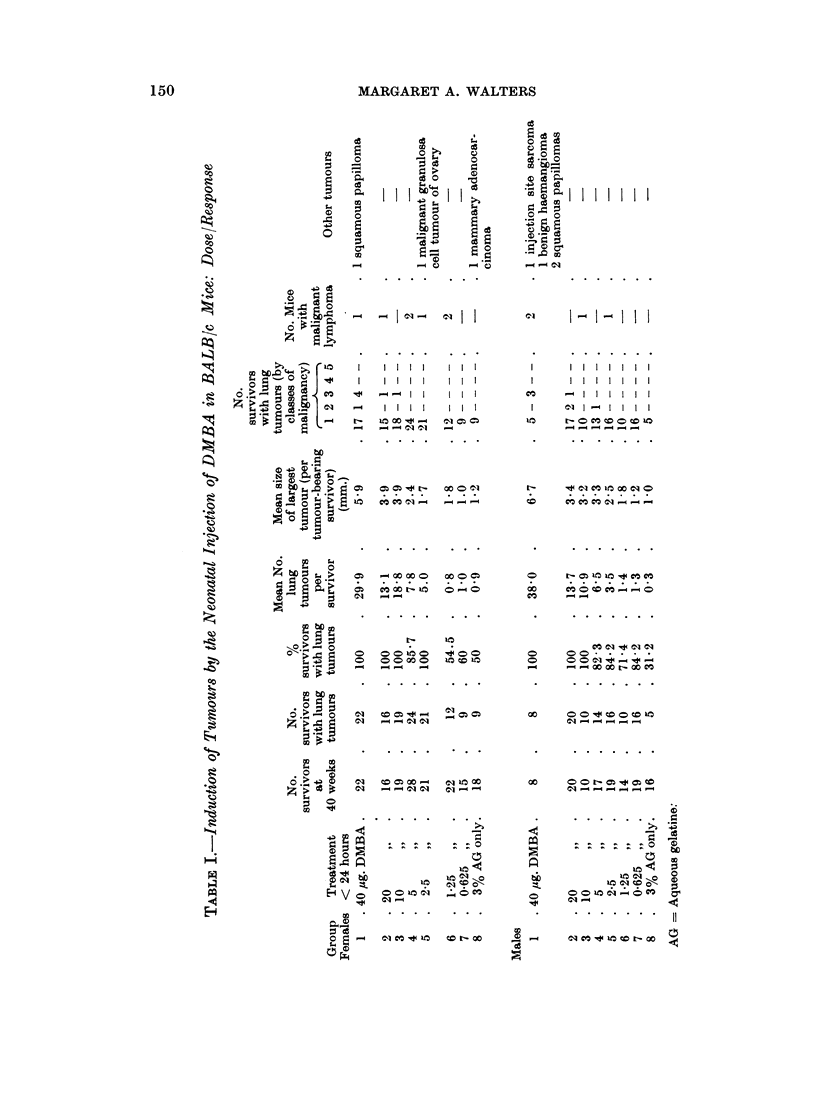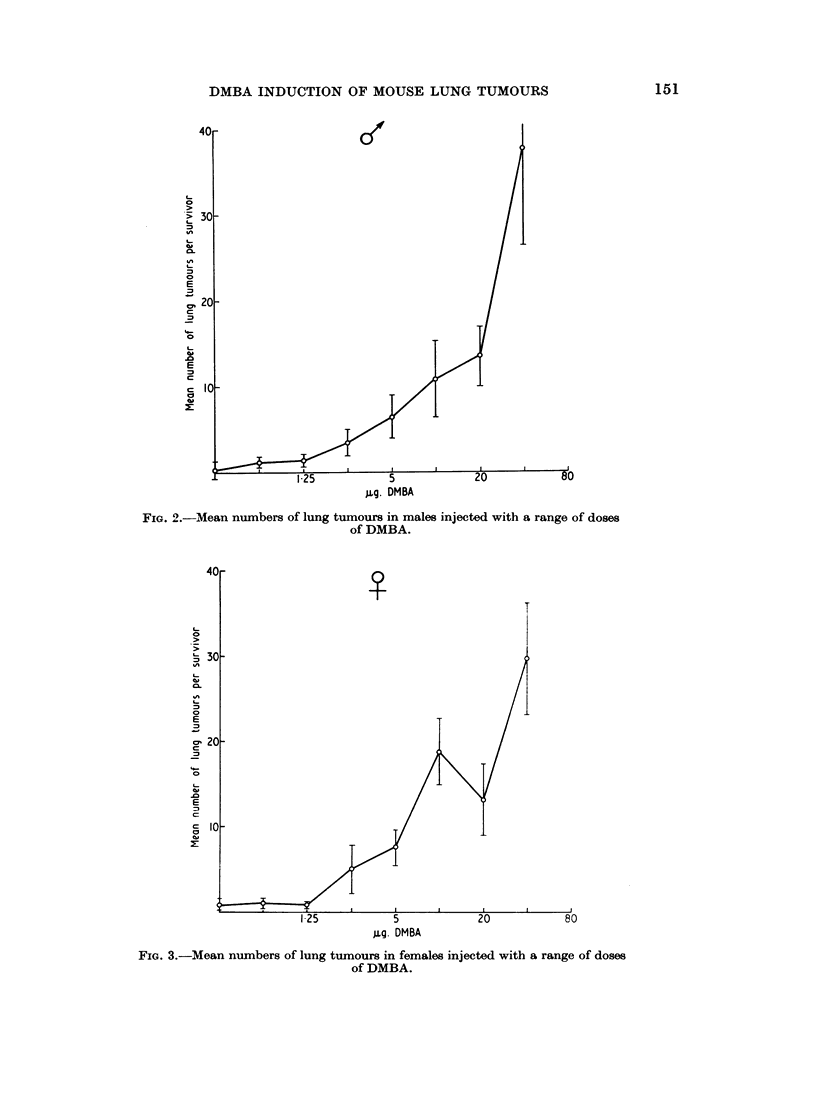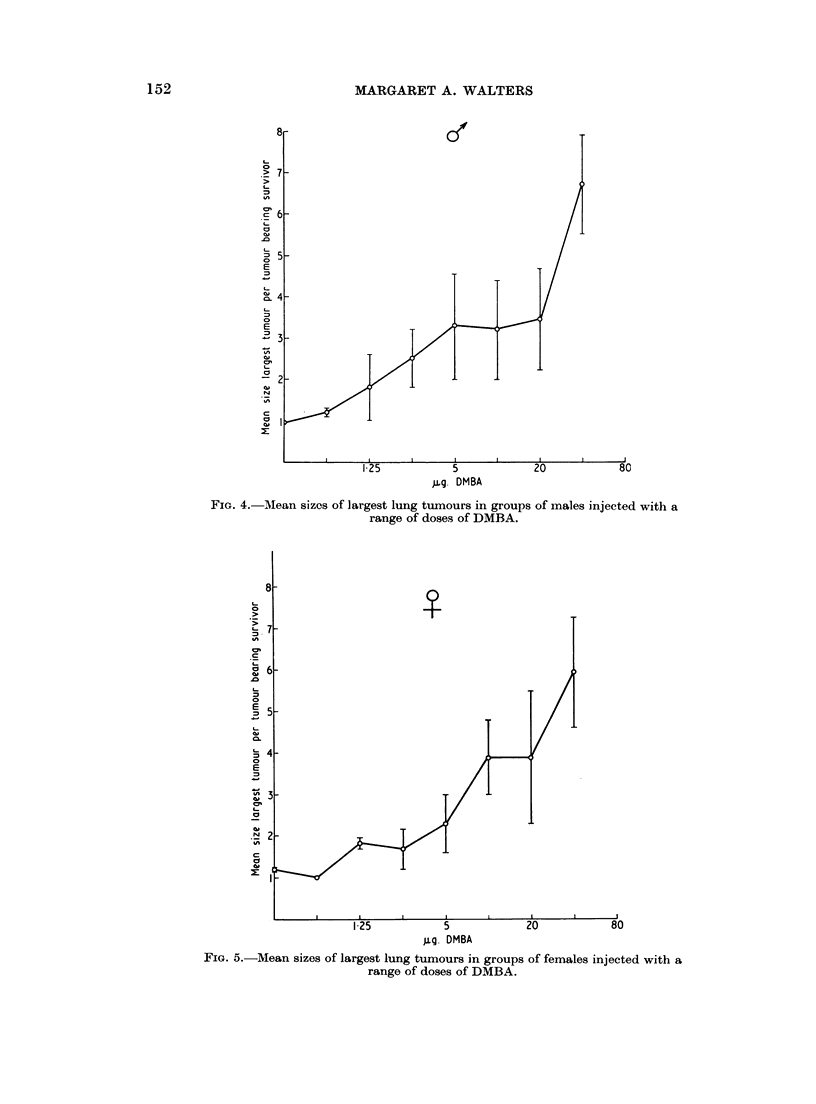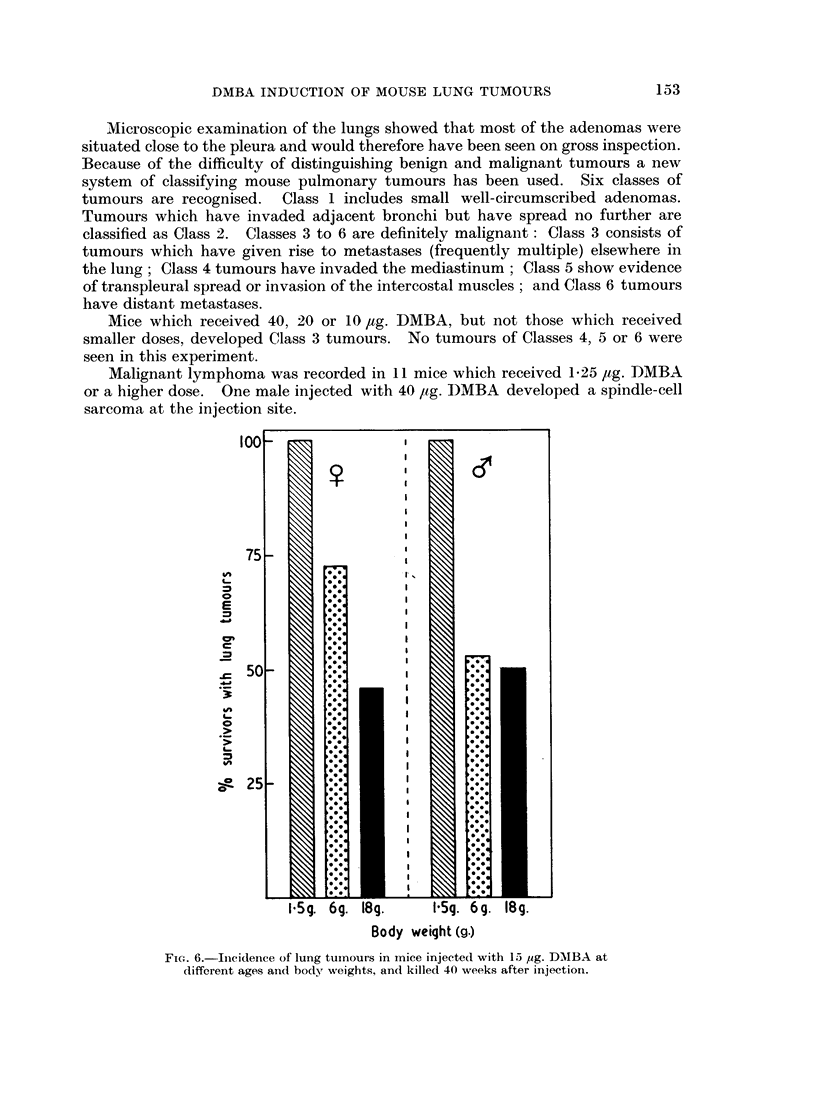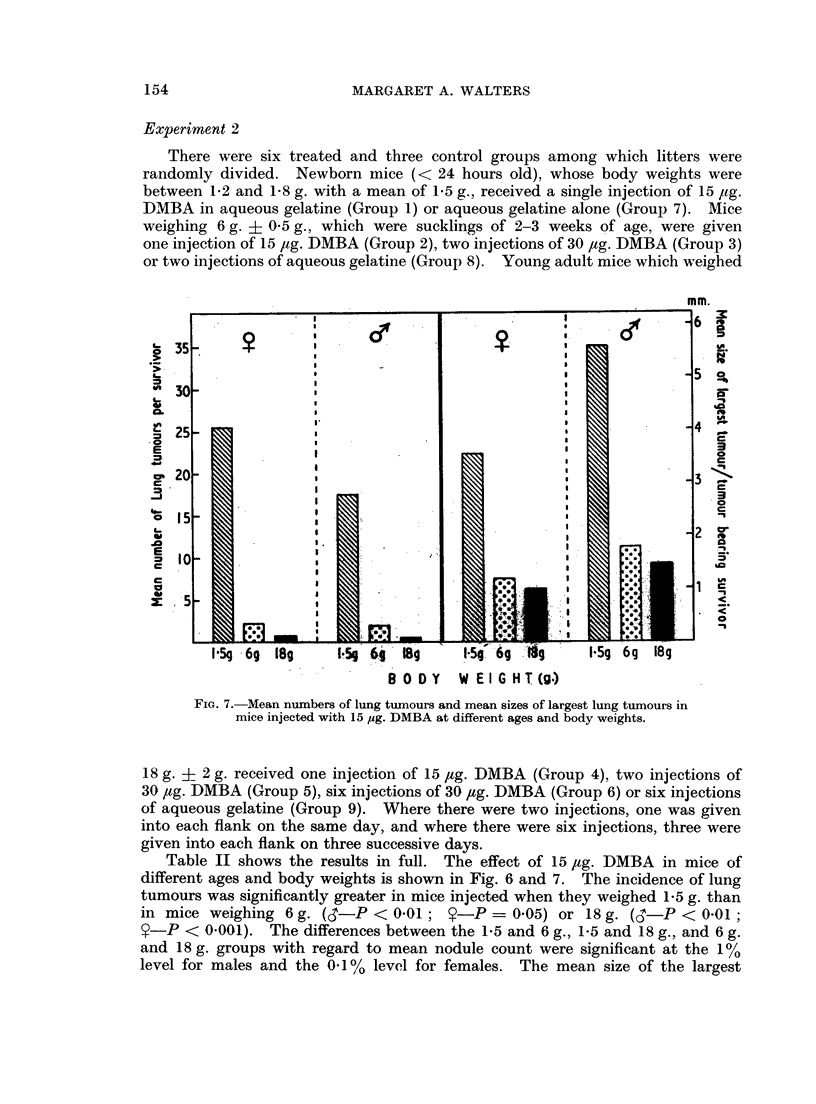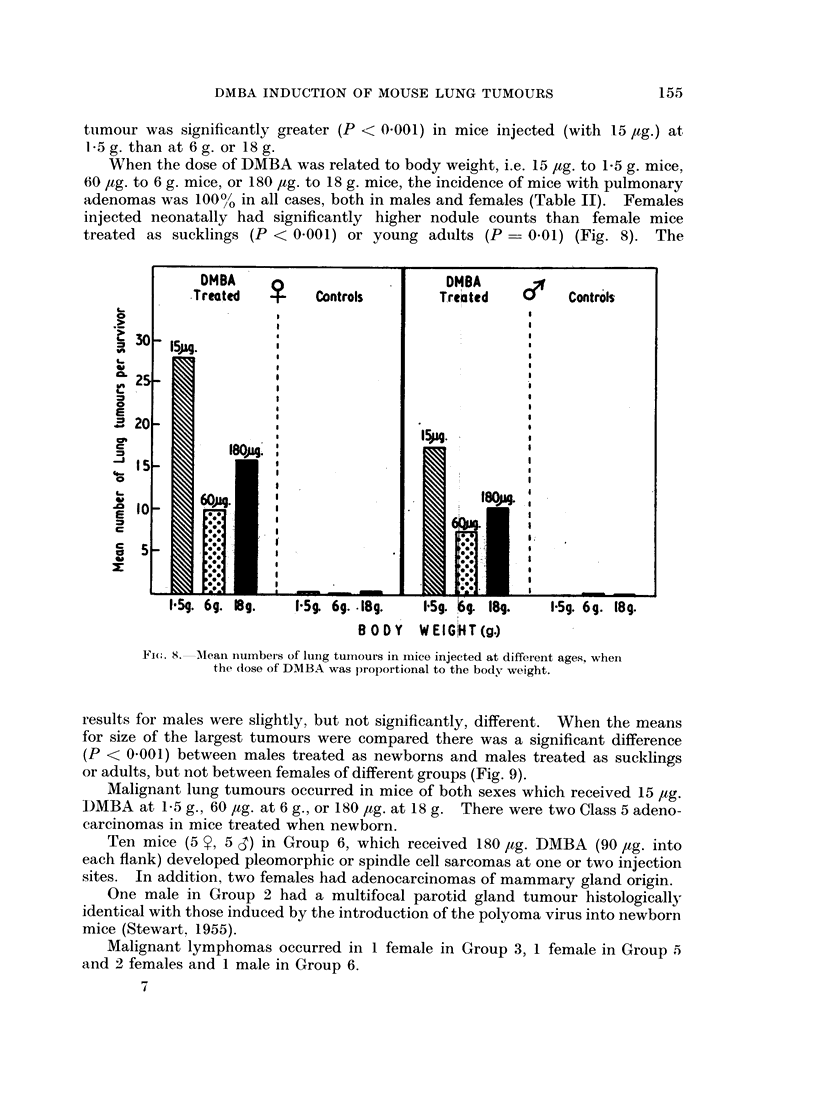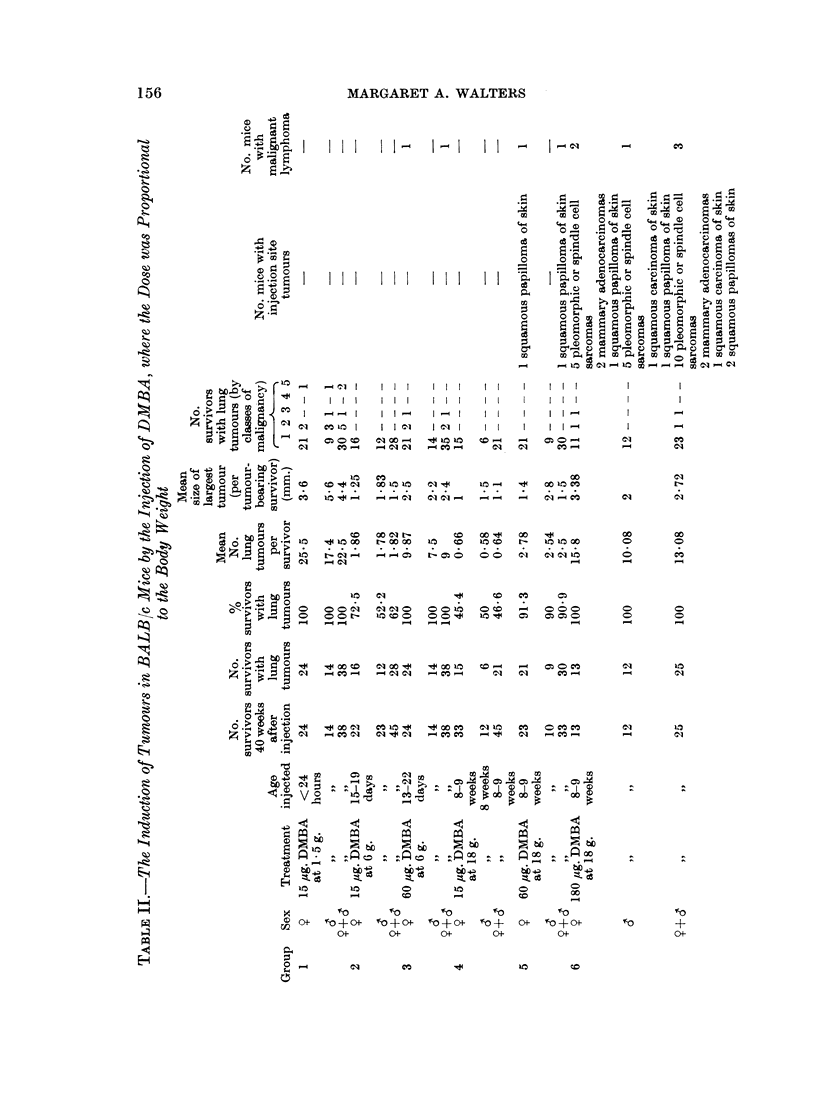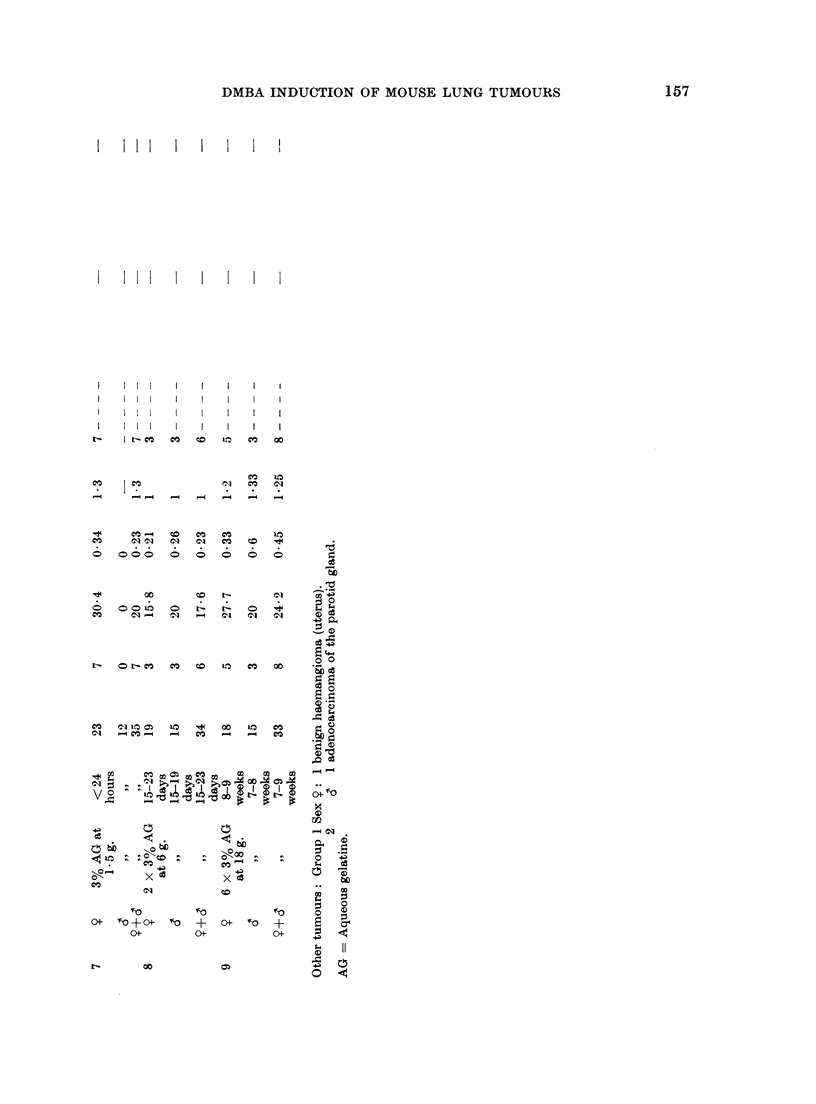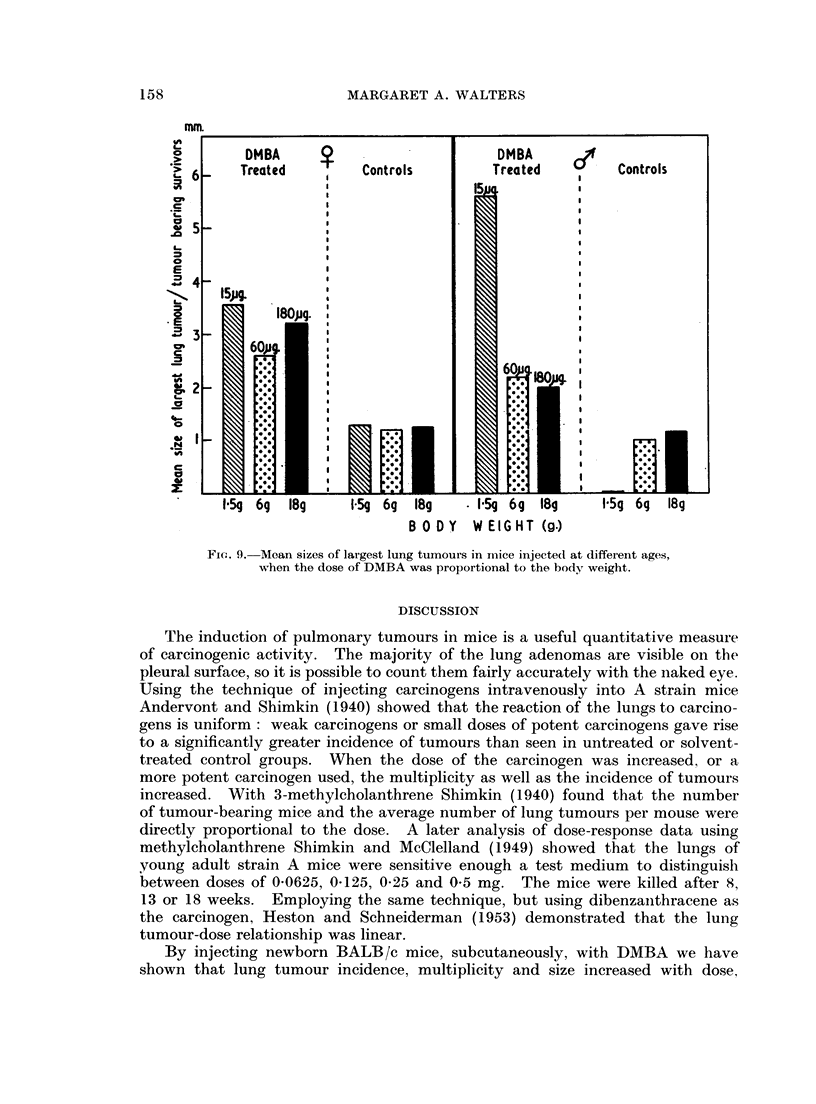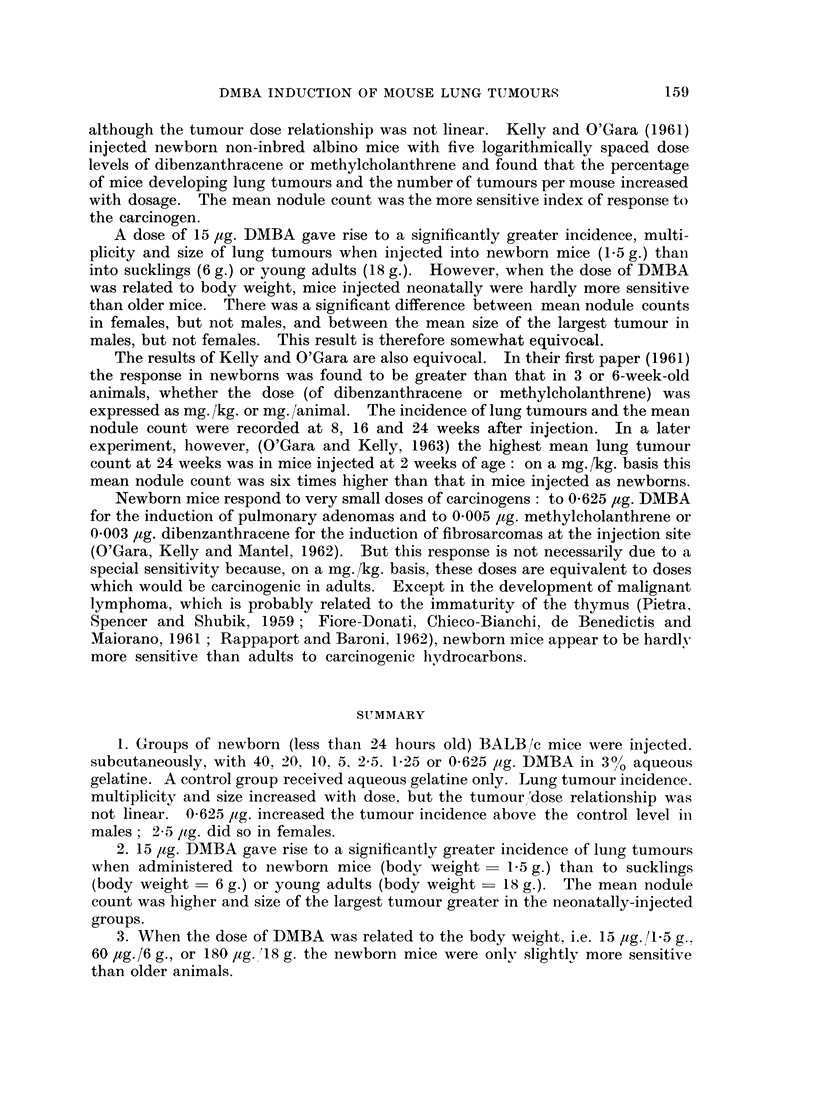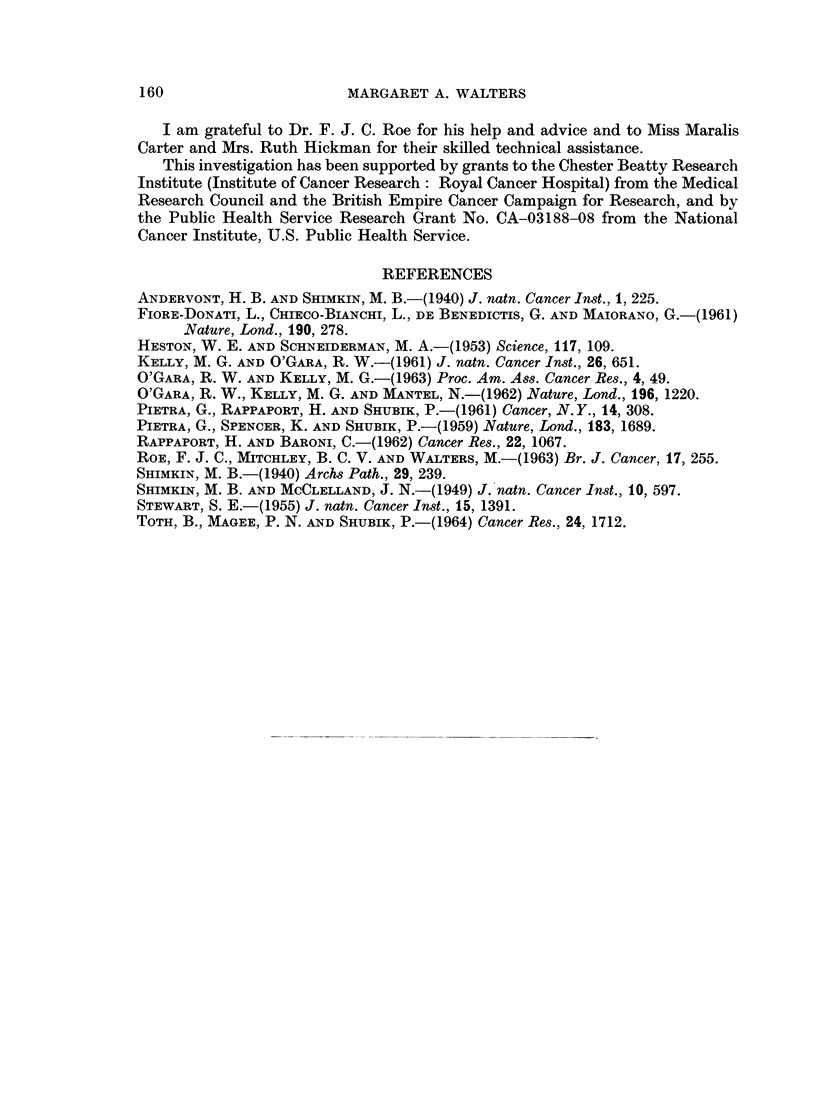# The induction of lung tumours by the injection of 9,10-dimethyl-1,2-benzanthracene (DMBA) into newborn suckling and young adult mice. A dose response study.

**DOI:** 10.1038/bjc.1966.17

**Published:** 1966-03

**Authors:** M. A. Walters


### THE INDUCTION OF LUNG TUMOURS BY THE INJECTION OF 9,10-DIMETHYL-1,2-BENZANTHRACENE (DMBA) INTO NEWBORN SUCKLING AND YOUNG ADULT MICE

### A Dose Response Study

MARGARET A. WALTERS

*From the Chester Beatty Research Institute, Institute of Cancer Research : Royal Cancer Hospital, Fulham Road, London, S.W.3*

Received for publication September 20, 1965

A VARIETY of neoplasms has been induced by the injection of polycyclic hydrocarbons, urethane, alkylating agents and nitrosamines into newborn mice (Pietra, Spencer and Shubik, 1959 ; Pietra, Rappaport and Shubik, 1961 ; Fiore-Donati, Chieco-Bianchi, de Benedictis and Maiorano, 1961 ; Roe, Mitchley and Walters, 1963 ; Toth, Magee and Shubik, 1964). The experiments to be described were designed to investigate the sensitivity of newborn mice with a view to using the technique for screening compounds for carcinogenic activity. A range of doses of DMBA has been tested for activity in newborn mice, and the sensitivity of the newborn to the induction of lung tumours has been compared with that of sucklings and young adults.

### MATERIALS AND METHODS

*Chemical agents.*—9,10-Dimethyl-1,2-benzanthracene (DMBA) was obtained from Roche Products Ltd. and gelatine powder from British Drug Houses. A suspension of DMBA in 3% aqueous gelatine was prepared by adding an acetone solution of the compound to aqueous gelatine warmed to 56° C. The acetone was driven off in a stream of nitrogen while the temperature was maintained at this level. The dose of DMBA administered to each mouse was contained in 0·02 ml. aqueous gelatine.

*Mice.*—BALB/c (Bittner agent free) mice were used in both experiments. The line was originally obtained from Dr. H. B. Andervont of the National Cancer Institute, Bethesda, Maryland, and has been maintained in this Institute by brother-sister mating since 1952. The mice were housed in metal cages and given water and fed a cubed diet (Diet 86, Messrs. Dixon and Sons, Ware, Herts.) *ad libitum*. Every mouse was vaccinated at 6 to 8 weeks of age as a precaution against ectromelia. Suckling and young adult mice were injected subcutaneously in the flank and newborn mice in the scapular region. In the case of the newborn, the needle was inserted at the root of the tail and the material was deposited in the scapular region to minimise leakage. Losses due to cannibalism by the mother were small.

The mice were weaned at 4 weeks, numbered on the ears and housed, 4–6 to a cage, according to group and sex. They were inspected every day, and examined thoroughly once a week, and sick mice were killed and autopsied. All surviving mice were killed 40 weeks after the day of injection and examined post mortem.

Adenomas on the surface of the lungs were counted and the diameter of the largest tumour was measured. All lung tumours larger than 3 mm. diameter and a proportion of the smaller adenomas were fixed in Bouin's fixative and sectioned. Lesions from other organs, which were definitely, or possibly neoplastic, were also taken for microscopical examination.

*Experiment* 1

Litters were allotted randomly to 8 groups. DMBA was administered to 7 groups in logarithmically spaced doses of 40, 20, 10, 5, 2·5, 1·25 and 0·625 μg. The control group received 0·02 ml. of 3% aqueous gelatine. All of the mice were injected when less than 24 hours old.

The results are presented in Table I. Response was measured by lung tumour incidence, the mean number of lung tumours per surviving mouse, and the mean size of the largest tumour per tumour-bearing survivor.

A dose as small as 0·625 μg. DMBA gave rise to a significantly greater incidence of lung tumours in males, as compared with the controls ($P < 0{\cdot}01$), but in females

FIG. 1.—Incidence of lung tumours in mice 40 weeks after neonatal injection with a range of doses of DMBA.

the difference was insignificant at doses smaller than 2·5 μg. (Fig. 1). The mean nodule count and mean size of the largest tumour increased with increasing dosages, but the tumour/dose relationship was not linear (Fig. 2–5). Differences in the mean number of tumours per survivor, significant at the 5% level, occurred between treated and control mice with a dose of 2·5 μg. in males and 10 μg. in females. There were too few controls with lung tumours to make a just comparison between the size of the largest tumours.

TABLE I.—*Induction of Tumours by the Neonatal Injection of DMBA in BALB/c Mice: Dose/Response*

| Group | Treatment < 24 hours | No. survivors at 40 weeks | No. survivors with lung tumours | % survivors with lung tumours | Mean No. lung tumours per survivor | Mean size of largest tumour (per tumour-bearing survivor) (mm.) | No. survivors with lung tumours (by classes of malignancy) 1 | 2 | 3 | 4 | 5 | No. Mice with malignant lymphoma | Other tumours |
|---|---|---|---|---|---|---|---|---|---|---|---|---|---|
| Females | | | | | | | | | | | | | |
| 1 | 40 μg. DMBA | 22 | 22 | 100 | 29·9 | 5·9 | 17 | 1 | 4 | – | – | 1 | 1 squamous papilloma |
| 2 | 20 ,, | 16 | 16 | 100 | 13·1 | 3·9 | 15 | – | 1 | – | – | 1 | — |
| 3 | 10 ,, | 19 | 19 | 100 | 18·8 | 3·9 | 18 | – | 1 | – | – | — | — |
| 4 | 5 ,, | 28 | 24 | 85·7 | 7·8 | 2.4 | 24 | – | – | – | – | 2 | — |
| 5 | 2·5 ,, | 21 | 21 | 100 | 5.0 | 1·7 | 21 | – | – | – | – | 1 | 1 malignant granulosa cell tumour of ovary |
| 6 | 1·25 ,, | 22 | 12 | 54.5 | 0·8 | 1·8 | 12 | – | – | – | – | 2 | — |
| 7 | 0·625 ,, | 15 | 9 | 60 | 1·0 | 1.0 | 9 | – | – | – | – | — | — |
| 8 | 3% AG only | 18 | 9 | 50 | 0·9 | 1·2 | 9 | – | – | – | – | — | 1 mammary adenocarcinoma |
| Males | | | | | | | | | | | | | |
| 1 | 40 μg. DMBA | 8 | 8 | 100 | 38·0 | 6·7 | 5 | – | 3 | – | – | 2 | 1 injection site sarcoma<br>1 benign haemangioma<br>2 squamous papillomas |
| 2 | 20 ,, | 20 | 20 | 100 | 13·7 | 3·4 | 17 | 2 | 1 | – | – | — | — |
| 3 | 10 ,, | 10 | 10 | 100 | 10·9 | 3·2 | 10 | – | – | – | – | 1 | — |
| 4 | 5 ,, | 17 | 14 | 82·3 | 6·5 | 3·3 | 13 | 1 | – | – | – | — | — |
| 5 | 2·5 ,, | 19 | 16 | 84·2 | 3·5 | 2·5 | 16 | – | – | – | – | 1 | — |
| 6 | 1·25 ,, | 14 | 10 | 71·4 | 1·4 | 1·8 | 10 | – | – | – | – | — | — |
| 7 | 0·625 ,, | 19 | 16 | 84·2 | 1·3 | 1·2 | 16 | – | – | – | – | — | — |
| 8 | 3% AG only | 16 | 5 | 31·2 | 0·3 | 1·0 | 5 | – | – | – | – | — | — |

AG = Aqueous gelatine.

FIG. 2.—Mean numbers of lung tumours in males injected with a range of doses of DMBA.

FIG. 3.—Mean numbers of lung tumours in females injected with a range of doses of DMBA.

FIG. 4.—Mean sizes of largest lung tumours in groups of males injected with a range of doses of DMBA.

FIG. 5.—Mean sizes of largest lung tumours in groups of females injected with a range of doses of DMBA.

Microscopic examination of the lungs showed that most of the adenomas were situated close to the pleura and would therefore have been seen on gross inspection. Because of the difficulty of distinguishing benign and malignant tumours a new system of classifying mouse pulmonary tumours has been used. Six classes of tumours are recognised. Class 1 includes small well-circumscribed adenomas. Tumours which have invaded adjacent bronchi but have spread no further are classified as Class 2. Classes 3 to 6 are definitely malignant : Class 3 consists of tumours which have given rise to metastases (frequently multiple) elsewhere in the lung ; Class 4 tumours have invaded the mediastinum ; Class 5 show evidence of transpleural spread or invasion of the intercostal muscles ; and Class 6 tumours have distant metastases.

Mice which received 40, 20 or 10 $\mu$g. DMBA, but not those which received smaller doses, developed Class 3 tumours. No tumours of Classes 4, 5 or 6 were seen in this experiment.

Malignant lymphoma was recorded in 11 mice which received 1·25 $\mu$g. DMBA or a higher dose. One male injected with 40 $\mu$g. DMBA developed a spindle-cell sarcoma at the injection site.

FIG. 6.—Incidence of lung tumours in mice injected with 15 $\mu$g. DMBA at different ages and body weights, and killed 40 weeks after injection.

*Experiment* 2

There were six treated and three control groups among which litters were randomly divided. Newborn mice (< 24 hours old), whose body weights were between 1·2 and 1·8 g. with a mean of 1·5 g., received a single injection of 15 $\mu$g. DMBA in aqueous gelatine (Group 1) or aqueous gelatine alone (Group 7). Mice weighing 6 g. $\pm$ 0·5 g., which were sucklings of 2–3 weeks of age, were given one injection of 15 $\mu$g. DMBA (Group 2), two injections of 30 $\mu$g. DMBA (Group 3) or two injections of aqueous gelatine (Group 8). Young adult mice which weighed

FIG. 7.—Mean numbers of lung tumours and mean sizes of largest lung tumours in mice injected with 15 $\mu$g. DMBA at different ages and body weights.

18 g. $\pm$ 2 g. received one injection of 15 $\mu$g. DMBA (Group 4), two injections of 30 $\mu$g. DMBA (Group 5), six injections of 30 $\mu$g. DMBA (Group 6) or six injections of aqueous gelatine (Group 9). Where there were two injections, one was given into each flank on the same day, and where there were six injections, three were given into each flank on three successive days.

Table II shows the results in full. The effect of 15 $\mu$g. DMBA in mice of different ages and body weights is shown in Fig. 6 and 7. The incidence of lung tumours was significantly greater in mice injected when they weighed 1·5 g. than in mice weighing 6 g. (♂—$P < 0{\cdot}01$ ; ♀—$P = 0{\cdot}05$) or 18 g. (♂—$P < 0{\cdot}01$ ; ♀—$P < 0{\cdot}001$). The differences between the 1·5 and 6 g., 1·5 and 18 g., and 6 g. and 18 g. groups with regard to mean nodule count were significant at the 1% level for males and the 0·1% level for females. The mean size of the largest tumour was significantly greater ($P < 0{\cdot}001$) in mice injected (with 15 $\mu$g.) at 1·5 g. than at 6 g. or 18 g.

When the dose of DMBA was related to body weight, i.e. 15 $\mu$g. to 1·5 g. mice, 60 $\mu$g. to 6 g. mice, or 180 $\mu$g. to 18 g. mice, the incidence of mice with pulmonary adenomas was 100% in all cases, both in males and females (Table II). Females injected neonatally had significantly higher nodule counts than female mice treated as sucklings ($P < 0{\cdot}001$) or young adults ($P = 0{\cdot}01$) (Fig. 8). The

FIG. 8.—Mean numbers of lung tumours in mice injected at different ages, when the dose of DMBA was proportional to the body weight.

results for males were slightly, but not significantly, different. When the means for size of the largest tumours were compared there was a significant difference ($P < 0{\cdot}001$) between males treated as newborns and males treated as sucklings or adults, but not between females of different groups (Fig. 9).

Malignant lung tumours occurred in mice of both sexes which received 15 $\mu$g. DMBA at 1·5 g., 60 $\mu$g. at 6 g., or 180 $\mu$g. at 18 g. There were two Class 5 adenocarcinomas in mice treated when newborn.

Ten mice (5 ♀, 5 ♂) in Group 6, which received 180 $\mu$g. DMBA (90 $\mu$g. into each flank) developed pleomorphic or spindle cell sarcomas at one or two injection sites. In addition, two females had adenocarcinomas of mammary gland origin.

One male in Group 2 had a multifocal parotid gland tumour histologically identical with those induced by the introduction of the polyoma virus into newborn mice (Stewart, 1955).

Malignant lymphomas occurred in 1 female in Group 3, 1 female in Group 5 and 2 females and 1 male in Group 6.

TABLE II.—*The Induction of Tumours in BALB/c Mice by the Injection of DMBA, where the Dose was Proportional to the Body Weight*

| Group | Sex | Treatment | Age injected | No. survivors 40 weeks after injection | No. survivors with lung tumours | % survivors with lung tumours | Mean No. lung tumours per survivor | Mean size of largest tumour (per tumour-bearing survivor) (mm.) | No. survivors with lung tumours (by classes of malignancy) | | | | | No. mice with injection site tumours | No. mice with malignant lymphoma |
|---|---|---|---|---|---|---|---|---|---|---|---|---|---|---|---|
| | | | | | | | | | 1 | 2 | 3 | 4 | 5 | | |
| 1 | ♀ | 15 $\mu$g. DMBA at 1·5 g. | <24 hours | 24 | 24 | 100 | 25·5 | 3·6 | 21 | 2 | – | – | 1 | — | — |
| | ♂ | ,, | ,, | 14 | 14 | 100 | 17·4 | 5·6 | 9 | 3 | 1 | – | 1 | — | — |
| | ♀+♂ | ,, | ,, | 38 | 38 | 100 | 22·5 | 4·4 | 30 | 5 | 1 | – | 2 | — | — |
| 2 | ♀ | 15 $\mu$g. DMBA at 6 g. | 15–19 days | 22 | 16 | 72·5 | 1·86 | 1·25 | 16 | – | – | – | – | — | — |
| | ♂ | ,, | ,, | 23 | 12 | 52·2 | 1·78 | 1·83 | 12 | – | – | – | – | — | — |
| | ♀+♂ | ,, | ,, | 45 | 28 | 62 | 1·82 | 1·5 | 28 | – | – | – | – | — | — |
| 3 | ♀ | 60 $\mu$g. DMBA at 6 g. | 13–22 days | 24 | 24 | 100 | 9·87 | 2·5 | 21 | 2 | 1 | – | – | — | 1 |
| | ♂ | ,, | ,, | 14 | 14 | 100 | 7·5 | 2·2 | 14 | – | – | – | – | — | — |
| | ♀+♂ | ,, | ,, | 38 | 38 | 100 | 9 | 2·4 | 35 | 2 | 1 | – | – | — | 1 |
| 4 | ♀ | 15 $\mu$g. DMBA at 18 g. | 8–9 weeks | 33 | 15 | 45·4 | 0·66 | 1 | 15 | – | – | – | – | — | — |
| | ♂ | ,, | 8 weeks | 12 | 6 | 50 | 0·58 | 1·5 | 6 | – | – | – | – | — | — |
| | ♀+♂ | ,, | 8–9 weeks | 45 | 21 | 46·6 | 0·64 | 1·1 | 21 | – | – | – | – | — | — |
| 5 | ♀ | 60 $\mu$g. DMBA at 18 g. | 8–9 weeks | 23 | 21 | 91·3 | 2·78 | 1·4 | 21 | – | – | – | – | 1 squamous papilloma of skin | 1 |
| | ♂ | ,, | ,, | 10 | 9 | 90 | 2·54 | 2·8 | 9 | – | – | – | – | — | — |
| | ♀+♂ | ,, | ,, | 33 | 30 | 90·9 | 2·5 | 1·5 | 30 | – | – | – | – | 1 squamous papilloma of skin | 1 |
| 6 | ♀ | 180 $\mu$g. DMBA at 18 g. | 8–9 weeks | 13 | 13 | 100 | 15·8 | 3·38 | 11 | 1 | 1 | – | – | 5 pleomorphic or spindle cell sarcomas<br>2 mammary adenocarcinomas<br>1 squamous papilloma of skin | 2 |
| | ♂ | ,, | ,, | 12 | 12 | 100 | 10·08 | 2 | 12 | – | – | – | – | 5 pleomorphic or spindle cell sarcomas<br>1 squamous carcinoma of skin<br>1 squamous papilloma of skin | 1 |
| | ♀+♂ | ,, | ,, | 25 | 25 | 100 | 13·08 | 2·72 | 23 | 1 | 1 | – | – | 10 pleomorphic or spindle cell sarcomas<br>2 mammary adenocarcinomas<br>1 squamous carcinoma of skin<br>2 squamous papillomas of skin | 3 |

| | | | | | | | | | | | | | | | |
|---|---|---|---|---|---|---|---|---|---|---|---|---|---|---|---|
| 7 | ♀ | 3% AG at 1·5 g. | <24 hours | 23 | 7 | 30·4 | 0·34 | 1·3 | 7 | – | – | – | – | — | — |
| | ♂ | ,, | ,, | 12 | 0 | 0 | 0 | — | – | – | – | – | – | — | — |
| | ♀+♂ | ,, | ,, | 35 | 7 | 20 | 0·23 | 1·3 | 7 | – | – | – | – | — | — |
| 8 | ♀ | 2 × 3% AG at 6 g. | 15–23 days | 19 | 3 | 15·8 | 0·21 | 1 | 3 | – | – | – | – | — | — |
| | ♂ | ,, | 15–19 days | 15 | 3 | 20 | 0·26 | 1 | 3 | – | – | – | – | — | — |
| | ♀+♂ | ,, | 15–23 days | 34 | 6 | 17·6 | 0·23 | 1 | 6 | – | – | – | – | — | — |
| 9 | ♀ | 6 × 3% AG at 18 g. | 8–9 weeks | 18 | 5 | 27·7 | 0·33 | 1·2 | 5 | – | – | – | – | — | — |
| | ♂ | ,, | 7–8 weeks | 15 | 3 | 20 | 0·6 | 1·33 | 3 | – | – | – | – | — | — |
| | ♀+♂ | ,, | 7–9 weeks | 33 | 8 | 24·2 | 0·45 | 1·25 | 8 | – | – | – | – | — | — |

Other tumours : Group 1 Sex ♀ : 1 benign haemangioma (uterus).

2 ♂ 1 adenocarcinoma of the parotid gland.

AG = Aqueous gelatine.

FIG. 9.—Mean sizes of largest lung tumours in mice injected at different ages, when the dose of DMBA was proportional to the body weight.

### DISCUSSION

The induction of pulmonary tumours in mice is a useful quantitative measure of carcinogenic activity. The majority of the lung adenomas are visible on the pleural surface, so it is possible to count them fairly accurately with the naked eye. Using the technique of injecting carcinogens intravenously into A strain mice Andervont and Shimkin (1940) showed that the reaction of the lungs to carcinogens is uniform : weak carcinogens or small doses of potent carcinogens gave rise to a significantly greater incidence of tumours than seen in untreated or solvent-treated control groups. When the dose of the carcinogen was increased, or a more potent carcinogen used, the multiplicity as well as the incidence of tumours increased. With 3-methylcholanthrene Shimkin (1940) found that the number of tumour-bearing mice and the average number of lung tumours per mouse were directly proportional to the dose. A later analysis of dose-response data using methylcholanthrene Shimkin and McClelland (1949) showed that the lungs of young adult strain A mice were sensitive enough a test medium to distinguish between doses of 0·0625, 0·125, 0·25 and 0·5 mg. The mice were killed after 8, 13 or 18 weeks. Employing the same technique, but using dibenzanthracene as the carcinogen, Heston and Schneiderman (1953) demonstrated that the lung tumour-dose relationship was linear.

By injecting newborn BALB/c mice, subcutaneously, with DMBA we have shown that lung tumour incidence, multiplicity and size increased with dose, although the tumour dose relationship was not linear. Kelly and O'Gara (1961) injected newborn non-inbred albino mice with five logarithmically spaced dose levels of dibenzanthracene or methylcholanthrene and found that the percentage of mice developing lung tumours and the number of tumours per mouse increased with dosage. The mean nodule count was the more sensitive index of response to the carcinogen.

A dose of 15 μg. DMBA gave rise to a significantly greater incidence, multiplicity and size of lung tumours when injected into newborn mice (1·5 g.) than into sucklings (6 g.) or young adults (18 g.). However, when the dose of DMBA was related to body weight, mice injected neonatally were hardly more sensitive than older mice. There was a significant difference between mean nodule counts in females, but not males, and between the mean size of the largest tumour in males, but not females. This result is therefore somewhat equivocal.

The results of Kelly and O'Gara are also equivocal. In their first paper (1961) the response in newborns was found to be greater than that in 3 or 6-week-old animals, whether the dose (of dibenzanthracene or methylcholanthrene) was expressed as mg./kg. or mg./animal. The incidence of lung tumours and the mean nodule count were recorded at 8, 16 and 24 weeks after injection. In a later experiment, however, (O'Gara and Kelly, 1963) the highest mean lung tumour count at 24 weeks was in mice injected at 2 weeks of age : on a mg./kg. basis this mean nodule count was six times higher than that in mice injected as newborns.

Newborn mice respond to very small doses of carcinogens : to 0·625 μg. DMBA for the induction of pulmonary adenomas and to 0·005 μg. methylcholanthrene or 0·003 μg. dibenzanthracene for the induction of fibrosarcomas at the injection site (O'Gara, Kelly and Mantel, 1962). But this response is not necessarily due to a special sensitivity because, on a mg./kg. basis, these doses are equivalent to doses which would be carcinogenic in adults. Except in the development of malignant lymphoma, which is probably related to the immaturity of the thymus (Pietra, Spencer and Shubik, 1959 ; Fiore-Donati, Chieco-Bianchi, de Benedictis and Maiorano, 1961 ; Rappaport and Baroni, 1962), newborn mice appear to be hardly more sensitive than adults to carcinogenic hydrocarbons.

### SUMMARY

1. Groups of newborn (less than 24 hours old) BALB/c mice were injected, subcutaneously, with 40, 20, 10, 5, 2·5, 1·25 or 0·625 μg. DMBA in 3% aqueous gelatine. A control group received aqueous gelatine only. Lung tumour incidence, multiplicity and size increased with dose, but the tumour/dose relationship was not linear. 0·625 μg. increased the tumour incidence above the control level in males ; 2·5 μg. did so in females.

2. 15 μg. DMBA gave rise to a significantly greater incidence of lung tumours when administered to newborn mice (body weight = 1·5 g.) than to sucklings (body weight = 6 g.) or young adults (body weight = 18 g.). The mean nodule count was higher and size of the largest tumour greater in the neonatally-injected groups.

3. When the dose of DMBA was related to the body weight, i.e. 15 μg./1·5 g., 60 μg./6 g., or 180 μg./18 g. the newborn mice were only slightly more sensitive than older animals.

I am grateful to Dr. F. J. C. Roe for his help and advice and to Miss Maralis Carter and Mrs. Ruth Hickman for their skilled technical assistance.

This investigation has been supported by grants to the Chester Beatty Research Institute (Institute of Cancer Research : Royal Cancer Hospital) from the Medical Research Council and the British Empire Cancer Campaign for Research, and by the Public Health Service Research Grant No. CA-03188-08 from the National Cancer Institute, U.S. Public Health Service.

### REFERENCES

ANDERVONT, H. B. AND SHIMKIN, M. B.—(1940) *J. natn. Cancer Inst.*, **1**, 225.
FIORE-DONATI, L., CHIECO-BIANCHI, L., DE BENEDICTIS, G. AND MAIORANO, G.—(1961) *Nature, Lond.*, **190**, 278.
HESTON, W. E. AND SCHNEIDERMAN, M. A.—(1953) *Science*, **117**, 109.
KELLY, M. G. AND O'GARA, R. W.—(1961) *J. natn. Cancer Inst.*, **26**, 651.
O'GARA, R. W. AND KELLY, M. G.—(1963) *Proc. Am. Ass. Cancer Res.*, **4**, 49.
O'GARA, R. W., KELLY, M. G. AND MANTEL, N.—(1962) *Nature, Lond.*, **196**, 1220.
PIETRA, G., RAPPAPORT, H. AND SHUBIK, P.—(1961) *Cancer, N.Y.*, **14**, 308.
PIETRA, G., SPENCER, K. AND SHUBIK, P.—(1959) *Nature, Lond.*, **183**, 1689.
RAPPAPORT, H. AND BARONI, C.—(1962) *Cancer Res.*, **22**, 1067.
ROE, F. J. C., MITCHLEY, B. C. V. AND WALTERS, M.—(1963) *Br. J. Cancer*, **17**, 255.
SHIMKIN, M. B.—(1940) *Archs Path.*, **29**, 239.
SHIMKIN, M. B. AND MCCLELLAND, J. N.—(1949) *J. natn. Cancer Inst.*, **10**, 597.
STEWART, S. E.—(1955) *J. natn. Cancer Inst.*, **15**, 1391.
TOTH, B., MAGEE, P. N. AND SHUBIK, P.—(1964) *Cancer Res.*, **24**, 1712.